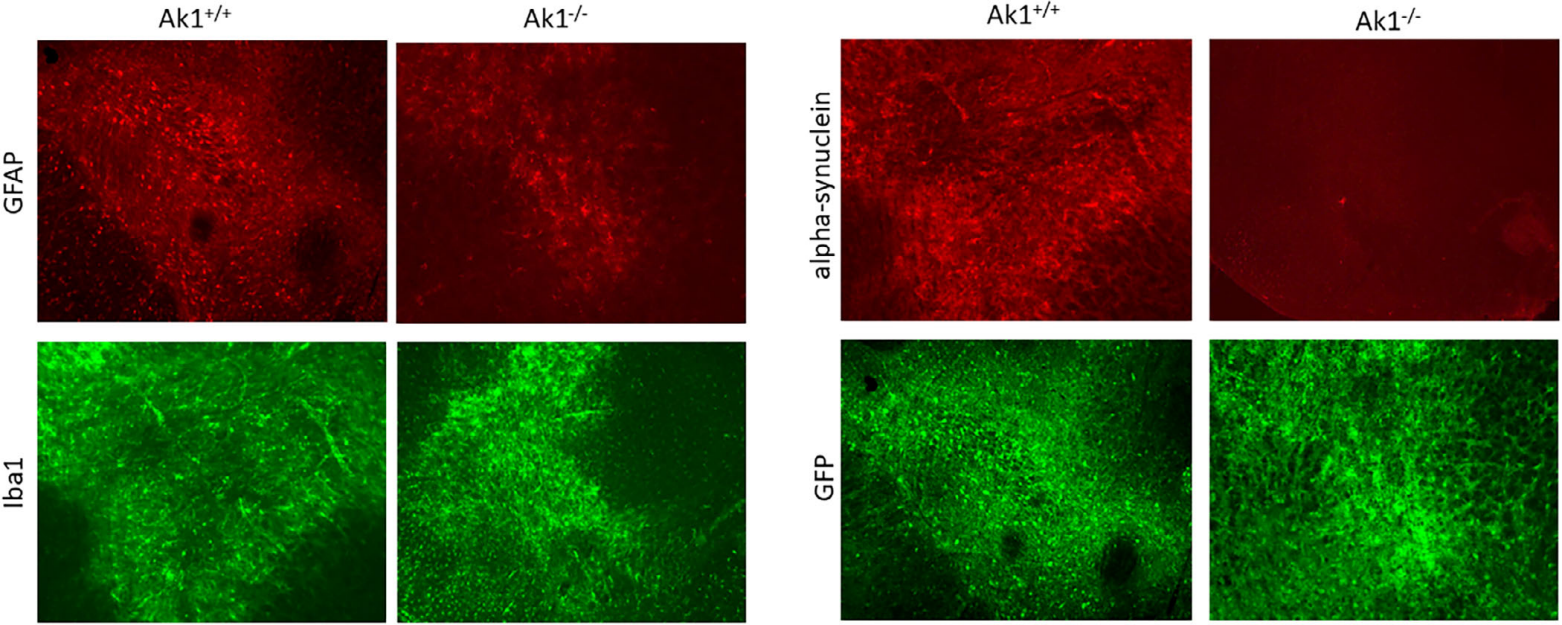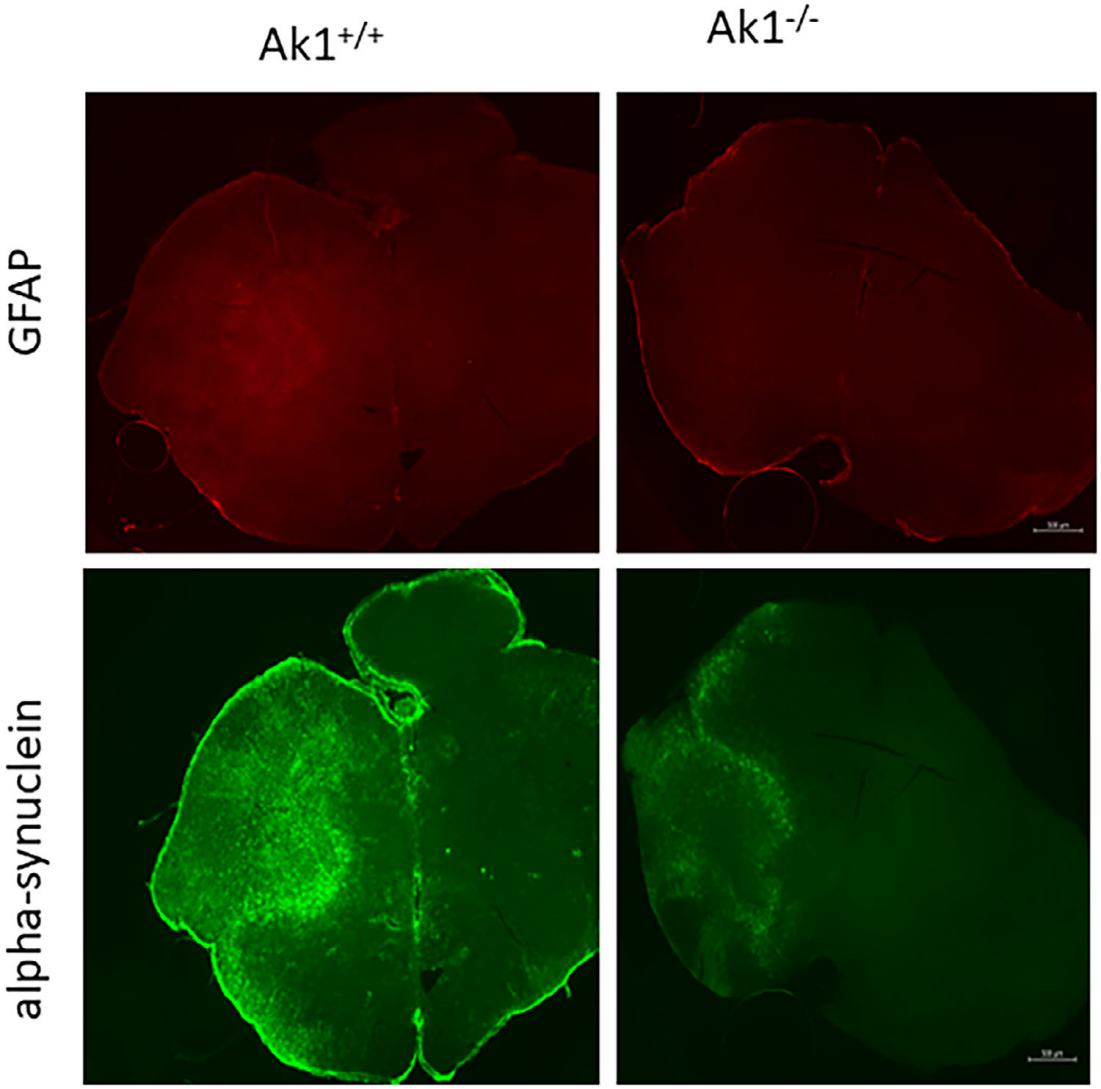# Investigating adenylate kinase 1 (Ak1) as a glial‐based therapeutic target in a mouse α‐synucleinopathy model

**DOI:** 10.1002/alz70855_103171

**Published:** 2025-12-24

**Authors:** Namrata Kumari, Maggie Sodders, Archana Marathi, Abby Olsen

**Affiliations:** ^1^ University of Pittsburgh, Pittsburgh, PA, USA

## Abstract

**Background:**

Despite dementia with Lewy bodies (DLB) being the second most prevalent cause of dementia and parkinsonism in the elderly, substantial progress in the development of disease‐modifying therapies remains elusive. Our preliminary investigations have shown that several α‐synucleinopathy risk genes are expressed in glial cells. We identified glial genes that either promote or inhibit α‐synuclein‐induced neurodegeneration in a large genetic screen in a *Drosophila* model of α‐synucleinopathy. In this screen, adenylate kinase 1 (Ak1) was a top hit, showing neuroprotective effects when knocked down in glial cells. Glial knockdown of Ak1 increased adenosine, and our primary hypothesis is that increasing adenosine is neuroprotective by way of reducing excitotoxicity. In the present study, we sought to determine if Ak1^‐/‐^ mice are protected in an alpha‐synucleinopathy model. This research will validate Ak1 as a therapeutic target in mammals.

**Method:**

We performed stereotaxic injection of AAV2‐α‐synuclein virus or AAV2‐GFP into 12‐week‐old C57BL/6 wild‐type and Ak1^‐/‐^. Total and phosphorylated alpha‐synuclein, reactive astrocytes, reactive microglia, dopaminergic neuron loss, and behavior were assessed at 1 month, 3 months, or 6 months post‐injection.

**Result:**

**Figure 1**: At the three‐month time point, preliminary data indicated a reduction in reactive astrocytes and α‐synuclein as demonstrated by decreased expression of GFAP along with lower α‐synuclein levels in Ak1‐/‐ mice compared to wild‐type mice. Image taken at 10x magnification; scale bar: 100 μm.

**Figure 2**: At the one‐month time point, preliminary data indicated a decrease in GFAP and α‐synuclein expression levels in Ak1^‐/‐^ mice compared to wild‐type mice. Image acquired at 2.5x magnification; scale bar: 500 μm.

**Conclusion:**

Our preliminary results indicate that Ak1^‐/‐^ mice have fewer reactive astrocytes and less total and phosphorylated α‐synuclein than their wild‐type littermates at 1 and 3 months post‐injection of AAV2‐α‐synuclein. These results support further investigation of Ak1 as a potential glial‐based therapeutic target in α‐synucleinopathies.